# Oral Nanoformulations in Cardiovascular Medicine: Advances in Atherosclerosis Treatment

**DOI:** 10.3390/ph17070919

**Published:** 2024-07-10

**Authors:** Xu Sun, Xushuang Jia, Zhaolin Tan, Dongmei Fan, Meiqi Chen, Ning Cui, Aidong Liu, Da Liu

**Affiliations:** School of Pharmacy, Changchun University of Chinese Medicine, Changchun 130117, China; nangongwentian@21cn.com (X.S.); jia07300909@163.com (X.J.); tzldj502@163.com (Z.T.); 15804440604@163.com (D.F.); 15567958349@163.com (M.C.); cuining_ccucm@163.com (N.C.)

**Keywords:** atherosclerosis, oral medicine, nanopreparations, new drug delivery systems

## Abstract

Atherosclerosis (AS) is the formation of atherosclerotic plaques on the walls of the arteries, causing them to narrow. If this occurs in the coronary arteries, the blood vessels may be completely blocked, resulting in myocardial infarction; if it occurs in the blood vessels of the brain, the blood vessels may be blocked, resulting in cerebral infarction, i.e., stroke. Studies have shown that the pathogenesis of atherosclerosis involves the processes of inflammation, lipid infiltration, oxidative stress, and endothelial damage, etc. SIRT, as a key factor regulating the molecular mechanisms of oxidative stress, inflammation, and aging, has an important impact on the pathogenesis of plaque formation, progression, and vulnerability. Statistics show that AS accounts for about 50 per cent of deaths in Western countries. Currently, oral medication is the mainstay of AS treatment, but its development is limited by side effects, low bioavailability and other unfavourable factors. In recent years, with the rapid development of nano-preparations, researchers have combined statins and natural product drugs within nanopreparations to improve their bioavailability. Based on this, this paper summarises the main pathogenesis of AS and also proposes new oral nanoformulations such as liposomes, nanoparticles, nanoemulsions, and nanocapsules to improve their application in the treatment of AS.

## 1. Introduction

Atherosclerosis (AS) is a pathological process triggered by arterial endothelial damage involving lipid deposition, macrophage phagocytosis, and foam cell formation. When present, pro-inflammatory factors lead to the formation of fibrous atherosclerotic lesions with a lipid core [1]. SIRT is a key factor regulating the molecular mechanisms of oxidative stress, inflammation, and aging. It is essential in the AS process, influencing plaque formation, progression, and vulnerability [2]. AS takes its name from its yellow appearance and the fact that it represents the beginning of a cardiovascular disease (CVD). With the changes in the spectrum of human diseases, AS is highly prevalent worldwide [3]. AS and the various diseases it causes, such as coronary artery disease, angina pectoris, heart attacks, and strokes, have become a leading cause of death [4].

Currently, AS treatments include the use of cholesterol-modifying drugs (e.g., statin, ezetimibe, and PCSK9 monoclonal antibodies) [5], anticoagulant drugs (e.g., rivaroxaban and warfarin) [6], antiplatelet drugs (e.g., aspirin and clopidogrel) [7], and healthy lifestyles (e.g., smoking cessation, sensible diet, and physical activity). However, these treatments have inevitable adverse effects. For example, statin drugs can induce myalgia, and antiplatelet drugs can cause bleeding [8]. Therefore, such treatments are very compromised when treating patients with AS [9]. Since AS has a long treatment period, is challenging to cure, and requires long-term medication to inhibit disease progression, oral delivery has become the main mode of drug delivery. That method can be used for the local or systemic administration of various drug molecules, from small molecules to biomolecules, and is often the most preferred therapeutic route [10].

Despite the obvious advantages of oral delivery, it remains challenging due to the human gastrointestinal tract’s complex environment. Conventional drugs for the treatment of atherosclerotic diseases suffer from high toxicity, low absorption, and inaccurate target localization, greatly limiting their clinical application [10,11]. Therefore, the pharmacological treatment of this condition has gradually become a research hotspot. 

In recent years, nanotechnology, as a new multifunctional technology, has demonstrated great advantages in AS treatment [12], including its more effective targeting [13], better bioavailability in diseased tissues [14], and reduced adverse effects. Therefore, it has become a potential new therapeutic tool for AS treatment in clinical practice. Particles in the nanometer size range are called nanoparticles (NPs). Nanomedicine uses NPs as carriers to deliver therapeutic drugs targeting specific cellular or tissue constituents. It is a precise method of treating diseases established at the molecular level. Several related reports have demonstrated that different sizes of nanomaterials can affect the targeting efficiency at the inflammation site. The particles’ size is central to their accumulation on the lesion site, and the composition of NPs may be of biological or chemical origin [15,16]. Nanoformulation of drugs overcomes common obstacles in the colon, such as the thick mucus layer, disrupted epithelial cells, and altered transit time, and can greatly improve drug bioavailability [17,18].

Studies have demonstrated that some new nanopreparations, such as NPs and nanoemulsions, can effectively improve the shortcomings of traditional medicines and reduce patients’ suffering from side effects. This paper reviews the mechanism of atherosclerotic diseases and the new preparations of oral medicines related to them, seeking to provide a reference for the oral treatment of AS.

## 2. AS Pathogenesis and Related Therapeutic Agents

According to the World Health Organisation (WHO), CVDs are the leading cause of death globally, and AS remains one of the most important causes of CVD, including heart disease and stroke. Studies have demonstrated an increasing prevalence of children and adolescents suffering from CVD [19,20]. According to the Progression of Early Subclinical Atherosclerosis study, AS is highly prevalent in middle-aged cohorts [21,22].

**Figure 1:**
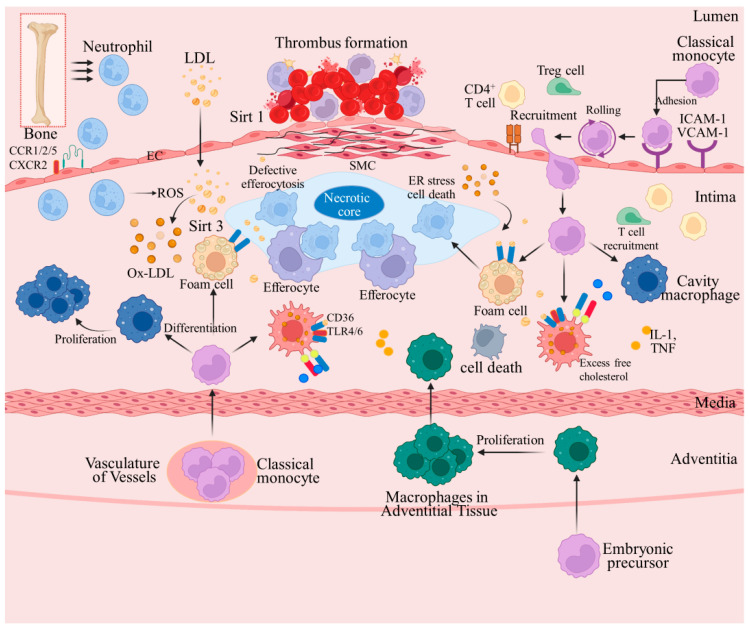
Mechanistic pathways and targets involved in AS pathogenesis.

AS is a chronic arterial vascular disease involving large and medium arteries, usually due to the deposition of cholesterol and other substances within the vessel wall, leading to inflammation and cholesterol plaque formation [23]. These events ultimately result in reduced vessel elasticity, vessel narrowing, and blood flow obstruction. The AS pathogenesis involves the complex action of multiple biological, biochemical, and physiological processes. In addition to traditional cardiovascular risk factors (e.g., diabetes mellitus, hypercholesterolemia, and smoking), nontraditional risk factors, including chronic diseases, genetics, and pregnancy-related complications, significantly increase the probability of developing CVD [24]. The pathogenesis can be divided into pre-mid and late stages, each with its own specific characteristics and pathogenesis (Figure 1).

### 2.1. Changes in Bioactive Substances Induced by Endothelial Cell Damage

The starting point of AS is usually endothelium damage. Endothelial cells (EC) [25] are a key component of the inner blood vessel wall, which is primarily responsible for maintaining the normal function and structure of the blood vessel and forming a natural barrier [26]. Factors like high cholesterol levels, high blood pressure, smoking, diabetes, inflammation, and immune response can trigger EC damage.

The interaction of oxidized low-density lipoprotein (ox-LDL) with endothelial cell receptor-1 (LOX-1) [27] and the expression of specific genes [28] (cholesteryl ester transfer protein) lead to the release of cytosolic molecules and increased oxidative stress, which increases the expression of vascular inflammatory factors, consequently triggering the aberrant function of vascular ECs. Additionally, pathologies like hyperaldosteronism, hypertension, and hyperglycaemia lead to oxidative stress and can damage ECs [29]. Diao et al. demonstrated that the accelerating effect of diabetes on AS could be attenuated by lowering blood glucose and serum lipid levels and enhancing the antioxidant capacity of ECs by inhibiting YTHDF2-mediated m6A modification of SIRT3 mRNA [30]. Additionally, Zhang et al. found that p-methylbenzenesulfonate (PCS) damages vascular ECs via the TLR4/TREM-1 pathway in an ApoE^−/−^ high-fat diet mouse model, which in turn promotes AS formation [31]. 

Physiological factors and poor lifestyle habits are central triggers for AS development. Chemicals in tobacco and nicotine can likewise damage ECs. Wu et al. also found that senescent vascular EC accumulation leads to chronic inflammation, which ultimately induces endothelial dysfunction and poses a risk of AS development and progression [32]. Anti-aging processes may become a novel strategy to prevent AS.

However, EC damage is not limited to physiological and environmental factors. The overactivation of inflammatory and immune responses may also lead to EC damage, exacerbating the inflammatory response in the vascular endothelium [33]. The release of multiple pro-atherosclerotic inflammatory factors, including molecular signals like TNF-α and interleukins IL-1β, IL-6, and IL-8, along with the increased expression of leukocyte adhesion molecules (e.g., VCAM-1 and ICAM-1), further increases the risk of plaque formation [34]. Studies on curcumin and fermented pu-erh tea have highlighted key mechanisms of inflammatory factor release and provided directions for exploring new pharmacological avenues of treatment [35,36].

SIRT1 is one of the most prominently expressed members of the Sirtuins family in the vascular system. SIRT1 maintains the normal physiological state of blood vessels by regulating the function of vascular ECs [37]. Nitric oxide (NO) production is essential for maintaining vascular function [38], and damage to ECs reduces NO production, leading to impaired vascular function [39]. A study by Mudau et al. found that NO is key in inhibiting pro-inflammatory cytokine secretion, immune cell extravasation, and thrombosis, which helps maintain the vascular wall’s homeostatic state [40]. SIRT1 is also expressed in vascular smooth muscle cells (VSMCs) and is involved in the modulation of vasodilatation and vasoconstriction. SIRT1 affects the vascular tone, maintaining the normal function of blood vessels. Increased expression of the SIRT1 protein increases eNOS activity and inhibits NOX-related oxidative stress, thus improving vascular endothelial dysfunction and vascular compliance [41]. Compounds like naringin (naringa) helped improve endothelial function in hypercholesterolemic rats by improving vascular function, increasing NO levels, and reducing the production of oxygen-free radicals [42]. They also helped decrease the expression levels of the oxidative stress-related proteins LOX-1, NADPH, and iNOS while attenuating the oxidative damage markers 3-NT and 4-HNE. This observation highlights the importance of ECs for vascular health and provides a potential therapeutic avenue for AS prevention.

### 2.2. Interaction between Lipid Deposition and Inflammatory Response

In the early AS stages, lipid deposition in the endothelium’s damaged areas, LDL infiltration and accumulation, particularly in damaged EC areas, and ox-LDL uptake by ECs create an inflammatory response in the foam cells, laying the groundwork for AS to develop.

Nur77 deficiency may increase NLRP3 inflammatory vesicle-mediated inflammation [43]; however, salbutamol B intervenes in AS and inflammation by regulating the NF-κB/NLRP3 pathway [44]. Rab27a activation promotes foam cell formation and inflammatory responses, contributing to uremic-accelerated AS progression through the NF-κB signalling pathway [45]. Additionally, diketone modification of Apo B-100 is a key factor contributing to vessel wall damage, AS, and endothelial dysfunction [46].

Under the effect of inflammation, ox-LDL gradually deposits in the damaged area to form lipid plaques. Li et al. demonstrated that ox-LDL induced apoptosis, inhibited cell proliferation and migration, and affected mitochondrial function [47]. Ox-LDL in macrophages induces oxidative stress, macrophage mitochondrial damage, and endoplasmic reticulum stress, with BTK tyrosine kinase being a key gene associated with oxidative stress, ER stress, and inflammation in macrophage-induced AS [48,49]. Studies have indicated that quercetin intake reduced lipid deposition in the arterial lumen and reduced serum sICAM-1, IL-6, and VCAM-1 levels in the aorta of ApoE^−/−^ mice, contributing to improved EC morphology and reduced apoptosis and reactive oxygen species (ROS) production [50,51]. In a rat metabolic model, combining atorvastatin + selenium NPs significantly increased SOD activity in the aorta, providing good protection against oxidative stress induced by lipid metabolism disorders [52]. EC damage and inflammatory response occur throughout AS pathogenesis; therefore, EC protection is crucial to prevent and treat AS.

### 2.3. Intermediate AS Stages

The middle stage of AS involves lipid deposition and an inflammatory response. After EC damage, lipids gradually accumulate to form plaques. Notably, the activation of branched-chain amino acid aminotransferase 1 exacerbates plaque formation by increasing ox-LDL-induced lipid accumulation and inflammatory responses [53]. Inflammatory cells, such as VSMCs [54], lymphocytes [55], monocytes, and macrophages [56], aggregate and release inflammatory mediators, creating a vicious cycle. PSRC1 deletion increases TMAO production and accelerates AS plaque formation [57].

NF-kB is a major regulator of inflammation [58]. The modulation of excessive inflammatory responses by NF-κB inhibition further reduces intravascular plaque formation. SIRT proteins inhibit the production of inflammatory factors and the development of inflammatory responses by regulating multiple signalling pathways. For example, SIRT1 inhibits the NF-κB signalling pathway, reducing the expression of inflammatory factors, such as IL-6 and TNF-α. Hou et al. found that the SREBP2-mediated NLRP3/ASC/Caspase-1 signalling pathway can induce inflammation and accelerate the AS process [59], promoting inflammation, increasing lipid uptake, and contributing to lipid deposition through the activation of the PI3K-AKT/MAPK/NF-κB signalling pathway [60]. The antimalarial drug efanamic acid [61] reduces inflammatory factor production and arterial plaque deposition by inhibiting the NF-κB-NLRP3 inflammatory pathway, thereby ameliorating AS. Overexpressing intracellular chloride channel 1 (CLIC1) accelerates plaque development, amplifies oxidative stress, and increases the release of inflammatory factors, while the inhibition of CLIC1 expression significantly reduces the expression of TNF-α, IL-1β, ICAM-1, and VCAM-1 [62].

Ox-LDL can cause endothelial dysfunction and iron death (ferroptosis), increasing lipid deposition and plaque area in atherosclerotic mice [63]. In addition to the modulation of biological processes, drugs like rosuvastatin attenuate the effects of a high-fat diet on AS and improve serum metabolic indices and IL6 and CCL2 levels [64]. Meanwhile, Palekar et al. [65] observed that treatment with antithrombin NPs carrying the thrombin inhibitor PPACK can reduce inflammation, exhibit plaque procoagulant activity, and slow plaque expansion. This treatment is expected to rapidly reduce the thrombotic risk after topical application.

Furthermore, polysorbate 407-induced AS leads to dyslipidemia, increased arterial wall thickness, collagen deposition, and elevated levels of inflammatory factors (IL-6 and TNF-α) [66]. Another study indicated that Yangmuin significantly ameliorates AS by affecting the migration and cholesterol efflux of THP-1 monocytes, further inhibiting the activation of the LXR/RXR pathway and angiogenesis, ultimately promoting the thromboxane-1-mediated effects of macrophages [67].

The arterial wall becomes swollen due to plaque formation, resulting in localized vessel obstruction. Typically, stable plaques are small and covered by a fibrous cap that is less likely to rupture. VSMC aging accelerates unstable plaque formation [68]. Recent findings indicate that ATP citrate lyase (Acly) is activated in inflammatory macrophages and AS plaques. In macrophages, inhibiting Acly results in plaques taking on a more stable character, as evidenced by an increase in collagen deposition and fibrous cap thickness with a concomitant reduction in necrotic cores [69]. Thus, modulating Acly activity may be a viable strategy to improve the prognosis of AS by helping stabilize plaque formation and slow down disease progression.

### 2.4. Advanced AS Stages

In the advanced AS stages, plaque rupture and thrombosis formation become key pathological processes. Factors like inflammation, TNF-α, matrix metalloproteinases, and others can make the fibrous caps (mainly made of collagen) within the plaque fragile, leading to plaque instability. Plaque rupture releases substances that promote thrombosis, and blocking MAO-B may help reduce arterial plaque by attenuating oxidative stress and inflammation while lowering blood triglyceride and LDL cholesterol levels [70]. SIRT1 downregulation may lead to blockage of anti-inflammatory and antioxidant pathways. In AS, decreased SIRT3 activity may lead to mitochondrial dysfunction and accelerated oxidative stress.

In vitro, METTL3-mediated m6A modification promoted ox-LDL-induced phenotypic transformation of VSMCs through the miR-375-3p/PDK1 axis, making plaques more susceptible to damage [71]. Additionally, damaged ECs can increase the expression of adhesion molecules, attracting more inflammatory cells (e.g., leukocytes), thus exacerbating plaque instability [72].

EC injury impacts NO release, leading to a decrease in the diastolic capacity of blood vessels, ultimately causing stenosis. EC damage and inflammatory responses may impact the entire structure of the vessel wall, including by increasing collagen and calcification, ultimately leading to vascular sclerosis and loss of elasticity. A previous study revealed the effect of *Fusobacterium nucleatum* on the course of AS through animal and in vitro co-culture models [72]. The results indicated that *F. nucleatum* could cause plaques to become unstable, as evidenced by increased infiltration of subepithelial macrophages, M1 polarisation, lipid deposition, and apoptosis. The invasion of this microorganism into aortic tissue significantly accelerated the progression of AS lesions.

Smooth muscle cells proliferate within the plaque, leading to increased plaque size and vessel narrowing. A study conducted by Han et al. indicated that HOXA1 promotes AS progression by activating NF-κB RelA (p65) and KLF4, increasing lipid deposition in VSMCs and the phenotypic conversion of macrophages [73]. After plaque rupture, the exposed plaque surface promotes platelet and coagulation protein aggregation and thrombus formation. At the same time, the long-term evolution of plaque leads to the progressive calcification of blood vessels, which in turn increases the risk of heart disease and stroke. A study by Sakamoto et al. revealed that CD163+ macrophages induced hyaluronan synthase expression via the NF-κB pathway, inhibiting the onset of vascular calcification and promoting the development of high-risk plaques [74].

Plaque rupture and thrombosis signify AS progression, which is the main mechanism leading to cardiovascular events, while stenosis and blood vessel hardening are the final results of AS development. AS can have a latency period of decades, and symptoms may not be apparent in its early stages but gradually appear as the disease progresses. The clinical consequences of AS include ischaemic heart disease, ischaemic stroke, and peripheral arterial disease [75], depending on the site of plaque formation and the degree and speed of vessel occlusion [9]. When AS progresses to an advanced stage, more severe clinical manifestations may occur. As these manifestations vary from one person to another, AS prevention and treatment usually require a combination of interventions, including lifestyle changes (e.g., healthy dietary and exercise habits), pharmacological treatments, and surgical interventions when necessary. Early prevention, regular medical check-ups, and maintaining a healthy lifestyle are essential to reduce the risk of AS-related diseases. Detailed information on AS pathogenesis, including the cell types involved, relevant targets, inflammatory factors or therapeutic pathways, damage location, and possible therapeutic agents, are presented in Table 1.

## 3. Novel Oral Nanoformulations for AS Treatment

**Figure 2:**
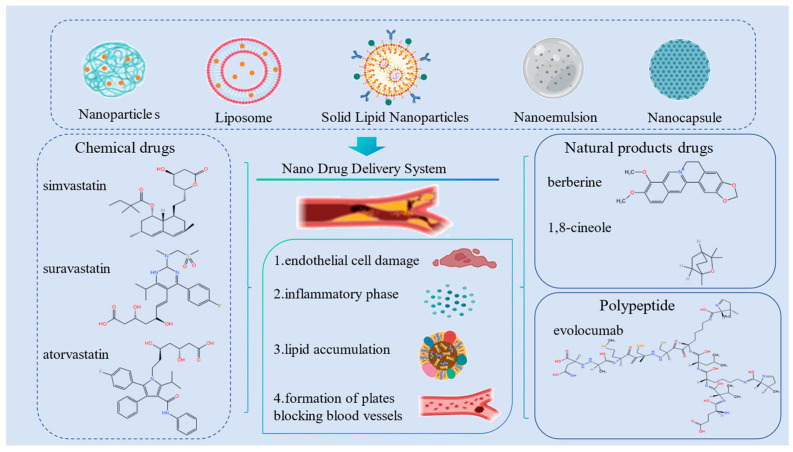
Main processes in AS pathogenesis and its treatment with oral nanoformulated drugs.

A nanodrug delivery system is a subparticulate drug carrier system that belongs to the microscopic category of nanoscales, including NPs, nanoliposomes, solid lipid NPs (SLNs), nanomicelles, nanoemulsions, nanocapsules, and nanogels [92,93,94,95]. Nanodrug delivery systems can encapsulate insoluble, fat-soluble, protein, nucleic acid, labile, or inactivated drugs [96,97,98]. Nanoformulations, which increase traditional drug solubility, increase the drug’s systemic circulation time and reduce its off-target cytotoxicity, reducing the required dose. They also increase the biofilm’s permeability, altering its distribution in the body and increasing its bioavailability to increase the drug’s stability [99,100,101]. Nanodrug carriers are widely used in medicine and hold great potential. Figure 2 presents oral nanoformulations and their associated therapeutic agents.

### 3.1. Oral NP-Loaded Drugs

NPs are solid colloidal nanosized particles (1–100 nm) made from natural or synthetic polymeric materials [102]. NPs are used as drug-carrying systems due to their ability to improve drug solubility [103], more accurately deliver drugs to target tissues [104], reduce side effects [105], and improve treatment effectiveness [106]. They can also improve the bioavailability of drugs and reduce adverse reactions [107]. They have gradually become a hotspot of medical research.

NPs can perform specific and nonspecific targeting in treating AS, migrating to the atherosclerotic lesion site through the ERP effect. At the same time, NPs can improve the targeting efficiency to diseased tissue based on the EPR effect [108,109]. In recent years, nanotechnology has been widely used in therapeutic studies on AS. Rosuvastatin has been reported to improve insulin sensitivity in a rat model by enhancing the expression of SIRT-1, PPAR-γ, and GLUT-4 in white adipose tissue [110]. Other studies have indicated that rosuvastatin can inhibit the activation of the NF-κB-p65 pathway and reduce the expression of IL-6, IL-8, intercellular adhesion molecule 1, and platelet endothelial cell adhesion molecule 1, thereby slowing down AS onset and progression [111]. Chen et al. [112] prepared rosuvastatin-chitosan NPs using the ionic gel method. A hypercholesterolemia model was induced with adult male rabbits, and lipids, IL-6 levels, and histopathology were examined. Rosuvastatin loaded with chitosan NPs had a significant hypolipidemic effect and inhibited the calcification of various valve tissues in experimental animals compared to rosuvastatin alone. Additionally, atorvastatin has a positive effect on AS treatment [18,113].

Statin (specifically atorvastatin and rosuvastatin) therapy may protect against CVD by inhibiting SIRT1 expression [114]. Atorvastatin enhances the expression of angiotensin II, inhibiting the expression of contractile proteins, such as α-SMA, SM-MHC, and SM22α, modulating the phenotypic transformation of VSMCs. At the same time, atorvastatin also prevents and treats AS by epigenetically regulating contractile proteins and mediating the phenotypic transformation of VSMCs through regulating the Akt/FOXO4 axis [115]. Liu et al. [116] developed atorvastatin-loaded polymer and lipid polymer hybrid NPs. Drug-loaded NPs exhibited better efficacy than a pure drug suspension in a healthy male Wistar rat model with induced hyperlipidaemia. The development of atorvastatin-loaded polymers and lipid-polymer hybrid NPs improves oral drug absorption and bioavailability while enhancing biopharmaceutical performance. They will help reduce the elevated lipid levels in hyperlipidaemia, which is the cause of AS, ultimately helping reduce the formation of AS plaques. Meanwhile, diosmin NPs, bindart-loaded NPs (pBIN), laminin-modified pBIN [117], bovine serum albumin, selenium NPs (SeNPs), surface-modified and chitosan (CS) surface-modified SeNPs [118,119], and sodium selenite [120] are central in AS treatment and are expected to be ready for clinical application soon.

The bioavailability of certain drugs for AS treatment is limited due to their low water solubility and rapid metabolism. NPs have a smaller size that allows them to cross cell membranes and the blood–brain barrier more easily, improving the distribution of the drug to the target tissue. This method makes the drug more targeted and reduces the impact on nontarget tissues, thus reducing side effects. However, the distribution and metabolism of NPs in the body may differ from the natural situation, which may cause potential toxicity and safety issues and susceptibility to aggregation and precipitation phenomena, which may affect the drug’s effectiveness and stability. Therefore, adequate research and evaluation are required to ensure the safety and efficacy of NPs.

### 3.2. Oral Drug-Loaded Nanoliposomes

Nanoliposomes are nanoscaled vesicles composed of lipid bilayers. They can improve drug bioavailability [121]. They increase the drug’s water solubility and stability by encapsulating it and enable targeted delivery, helping reduce unwanted effects on healthy tissues and side effects [122,123].

Nanotechnology emerged in the pharmaceutical field in the early 2000s, and some early studies focused on the feasibility of nanoliposomes for AS treatment [124]. In 2010, research on nanoliposomes to treat AS gradually increased, and researchers began to explore the mechanisms related to AS, such as the reduction of vascular inflammation and cholesterol deposition in the arterial wall by nanoliposomes [125]. Nanoliposome mechanisms in AS treatment include improved drug delivery, targeting, antioxidant effects, drug stability, reduced toxicity, improved bioavailability, and a controlled release rate [126,127,128,129,130]. These mechanisms work synergistically to help improve AS treatment.

In recent years, the use of nanoliposomes in AS research has continued to increase. Berberine (BBR) promotes autophagy in peritoneal macrophages by activating SIRT1 via the NAD+ synthesis pathway, promoting nuclear translocation and deacetylation of transcription factor EB. Its functional modulation may be a potential therapeutic strategy for AS treatment [131]. Other studies have claimed that BBR is one of the most promising natural products, with significant benefits for lipid and glucose metabolism. It stimulates KLF16 expression, which stimulates PPARα activation and enhances the interaction between KLF16 and PPARα to attenuate AS in diabetes mellitus [132]. Duong et al. [133] designed and generated pre-liposomes (PLs) loaded with BBR as a solid template for high-dose liposomes, thereby improving the oral bioavailability and therapeutic efficacy of BBR.

The pharmacokinetics and endogenous cholesterol-lowering effects of recombinant BBR liposomes were also investigated in Wistar male rats and mice. The oral bioavailability of BBR-containing liposomes in rats was significantly higher than that of pure BBR (oral suspension) liposomes. This study overcame the limitations associated with the poor oral bioavailability of BBR and is of great significance for the development of novel oral liposomes to treat hyperlipidaemia, thereby reducing the risk of AS.

Additionally, peptide PCSK9 antibody inhibitors can also be used in AS treatment [134]. D’Onofrio et al. demonstrated that PCSK9 antibody inhibitors have intrinsic anti-inflammatory, anti-autophagic, and antioxidant properties in ECs and that these pleiotropic effects may be mediated by SIRT3. They also revealed that SIRT3 may be a mediator of this pleiotropic effect [135]. Li et al. [136] designed an injectable bionic nanoliposome loaded with the PCSK9 inhibitor evolocumab to alleviate AS. This injectable evolocumab biomimetic nanoliposome demonstrates the potential for oral AS treatment, providing patients with more convenient treatment options through improved bioavailability and targeting. This technology is expected to be an essential component of innovative oral therapeutic options, reducing the burden of treatment for patients.

Nanoliposomes have excellent potential as a nanodrug delivery system for AS treatment. They can improve drug solubility, enhance bioavailability, enable targeted delivery, and reduce side effects. However, their preparation complexity, potential toxicity, and stability issues need to be further investigated and addressed.

### 3.3. Oral Drug-Loaded Solid Lipid NPs

SLNs are nanoscaled drug delivery systems comprising solid lipids as their core material, stabilized and encapsulated using surfactants to form particles of 10–1000 nm [137].

Compared to other nanodrug delivery systems, SLNs have better stability and are less prone to agglomeration or precipitation, keeping the NPs dispersed [33]. The potential advantages of SLNs in drug delivery include improving drug bioavailability, controlling the release rate, improving drug stability, and reducing toxicity [34,35,36]. Introduced in recent years to study AS treatment, SLNs can be used to deliver antioxidants, anti-inflammatory agents, modulatory drugs, and other drugs [138,139]. Research on oral SLNs for AS treatment is a decade old.

On the other hand, Zhang et al. [140] investigated and designed simvastatin (SV)-loaded SLNs comprising HS-15 or Tween 20 and oleic acid using an emulsified solvent evaporation technique. The study was conducted on male SD rats (180–200 g) and male ICR mice (18–20 g) maintained at 37 °C for enteral perfusion. The study’s results indicated that the oral bioavailability of SV doped with SLNs and the oral bioavailability of SVA were significantly increased compared to free SV in rodents. This study fills the gap in research related to enhanced oral bioavailability of SV by SLNs and presents SLNs as a promising drug delivery system to enhance oral SV bioavailability.

SV reduces AS by reducing oxidative stress, inhibiting the TGF-β/Smad signalling pathway, and inactivating Snail-1 and Twist-1 to inhibit endothelial–mesenchymal transition induced by 1-palmitoyl2-(5-oxovaleroyl)-sn-glycerol-3-phosphorylcholine [141]. Meanwhile, TNF-α is an important risk factor for AS, and SV can reduce TNF-α-induced apoptosis in ECs, where SIRT1 may play a key role in apoptosis [142]. Similarly, Rizvi et al. [143] studied and developed SV-loaded SLNs (SIM-SLNs). SIM-SLNs were prepared by nano-template engineering using palmitol as a lipid nucleus and Tween 40/Span 40/Myrj 52 as a stabilizing nucleus. SIM-SLNs were orally administered to male SD rats to observe their in vivo pharmacokinetics and anti-hyperlipidaemic activity. SIM-SLNS significantly reduced elevated blood lipids and decreased total cholesterol levels compared to control rats and SIM-dispersed treated hyperlipidaemic rats. That study demonstrated that orally administering SIM-SLNs significantly reduced hyperlipidaemia by lowering serum total cholesterol and non-HDL-C levels.

Nanostructured lipid carriers (NLCs) are a novel nanodrug delivery system comprising a mixture of solid and liquid lipids. NLCs have applications in various fields, particularly in treating CVDs. To improve the solubility and bioavailability of the oral lipid-lowering drug ezetimibe (EZ), researchers used ultrasound technology to prepare EZ-loaded nanostructured lipid carriers (EZ-NLC). After optimization, the resulting EZ-NLC displayed significant lipid-lowering effects in a high-fat diet-induced hyperlipidaemia rat model [144]. Researchers used 64Cu-labeled NLCs to verify their accumulation in the atherosclerotic plaques of ApoE^−/−^ mice, and NLCs demonstrated good safety in biocompatibility studies [145,146]. NLCs, as novel drug carriers, have the potential to enhance drug efficacy, improve targeting, and enhance biocompatibility, especially in treating and diagnosing diseases like AS. Through various surface modifications and optimization strategies, NLCs can more effectively deliver drugs to pathological sites, reduce side effects, and improve therapeutic outcomes.

Since hyperlipidaemia is a major risk factor associated with AS and subsequent heart disease, SIM-SLNS may be a promising approach for enhancing the therapeutic potential and reducing the dose of currently available oral formulations of SIM, which could have a very positive effect on AS prevention.

### 3.4. Oral Drug-Loaded Nanoemulsions

Nanoemulsions are emulsions with nanoscaled particles in which lipids, proteins, or other components are dispersed in water or other media [147]. Due to their small size, they can be more easily absorbed and utilized by biological systems. This helps increase the bioavailability of the drug or nutrient, enhancing its efficacy or absorption [148]. At the same time, nanoemulsions can be surface-modified for targeted delivery, reducing unwanted effects on healthy tissues [149].

During drug development, controlled release can be achieved by adjusting the structure and composition of the nanoemulsion so that the drug or other active ingredient is released at a specific rate, providing a longer-lasting effect [150,151]. Although nanoemulsions have many advantages, they also face issues of drug stability and toxicity assessment and regulation [152]. Therefore, developing and applying nanoemulsions requires detailed research and evaluation to ensure their safety and efficacy. Early research from the 1960s to the 1980s focused on colloid and interface science [153,154]. In the 1990s and early 2000s, researchers began to explore the potential of nanoemulsions as drug delivery systems. In recent years, research on preventing and treating AS has also begun to emerge due to the drug-carrying advantages of nanoemulsions [155,156]. Ahsan et al. [157] prepared a calcium-cured self-nanoemulsifying delivery system for rosuvastatin to improve its in vitro dissolution. The study used male albino rats of 150–200 g to induce hyperlipidaemia. The calcium-cured self-nanoemulsifying drug delivery system has great potential in improving the oral absorption of this insoluble drug and its pharmacodynamic effects.

In addition to rosuvastatin, 1,8-cineole (CIN) has been used for AS treatment. CIN reduces vascular tissue damage by lowering lipid parameters and inhibiting the expression of inflammatory factors and proteins, ultimately reducing the extent of atherosclerotic lesion areas [158]. Chen et al. [159] prepared oral nanoemulsions stabilized by polysaccharide–protein/protein complexes using microjet and UV irradiation to promote the therapeutic effect of CIN on AS. In this study, a nanoemulsion was formulated using a polysaccharide–protein/protein complex (dextran–bovine serum albumin/fish albumin) as an emulsifier and vitamin B12 as a ligand to facilitate transport through the small intestine. The nanoemulsion was also validated in vivo using an atherosclerotic mouse model. It was found to have significant anti-AS efficacy. These nanoemulsions also improved the ex vivo and in vivo stability of CIN, prolonged its retention time in the gastrointestinal tract, enhanced CIN permeability through the mucus layer and intestinal ECs, and increased CIN oral bioavailability and plaque accumulation. These results provide a promising oral formulation of essential oils with significant implications for AS treatment.

Nanoemulsions can increase drug solubility and stability relative to ordinary formulations, improving drug bioavailability and making it easier to be absorbed [160,161]. They also improve the tissue distribution and metabolism of the drug and reduce its accumulation in nontarget tissues, reducing the risk of side effects [162,163]. Oral nanoemulsions are, therefore, potentially advantageous in AS treatment but display issues of preparation complexity, potential toxicity, and stability issues that require further research and evaluation to determine their effectiveness and safety in clinical applications.

### 3.5. Oral Drug-Loaded Nanocapsules

A nanocapsule is a nanoscaled drug delivery system comprising a capsule with a core drug encapsulated inside it by a wall material [164]. Nanocapsules are designed to efficiently deliver drugs, compounds, or other active ingredients to target tissues or cells to enhance the therapeutic efficacy and reduce adverse drug reactions [165,166]. During drug loading, the nanocapsules protect the core drug or compound, thus improving drug stability and bioavailability [167,168]. They also enable the drug to be delivered more precisely where it is needed, reducing damage to healthy tissue and toxic side effects [169,170].

Early studies on nanocapsules as drug delivery systems date back to the late 20th and early 21st centuries. These studies have mainly focused on the concepts and technologies of nanocapsules as drug-delivery systems [171]. In AS treatment, tissue-targeted anti-inflammatory therapy might be a better option, promoting the regression of atherosclerotic plaques. Therefore, Matheus et al. developed a novel type of anti-PECAM-1 surface-functionalized metallic composite multi-wall nanocapsule containing a natural ω-3 fatty acid, DHA, as the lipid core. These nanocapsules are suitable as a drug delivery system for targeted therapy of inflamed endothelial tissues [172]. With a deeper understanding of the causes and mechanisms of AS, researchers are beginning to explore the potential of nanocapsules for AS treatment and prevention [173,174]. Nanocapsules can be used to deliver anti-inflammatory drugs, antioxidants, cholesterol modulators, and other medications to reduce plaque formation and arterial inflammation [172,175]. They can also be engineered to have targeted delivery properties to help deliver drugs accurately to atherosclerotic plaques [176]. Additionally, nanocapsules can be used as medical imaging agents to better diagnose and assess the extent of AS [177]. Currently, the main mode of drug delivery is oral. Because of the slow development of AS, the treatment cycle is long; AS is difficult to completely eradicate, and long-term medication is needed to inhibit its progression. Therefore, in recent years, oral nanocapsules have been increasingly researched.

MK-0616 is a potent oral PCSK9 macrocyclic peptide inhibitor that reduces the risk of AS by lowering LDL-cholesterol, non-HDL-cholesterol, apoB, and Lp(α) [178]. Low-dose curcumin encapsulated in hyaluronic acid-based nanocapsules exhibited antihypertensive effects in hypertensive rats. The study indicated that using hyaluronic acid-based nanocapsules could improve the bioavailability and efficacy of hydrophobic compounds like curcumin, offering a new potential method for targeted vascular therapy in hypertension [179].

Salaheldin et al. [180] developed the first small molecule oral nanomedicine targeting the liver for PCSK9 inhibition using nanotechnology methods. They synthesized and characterized a stable aqueous dispersion of a 150–200 nm nanocarrier-encapsulated drug (named P-4) through high-throughput optimization and a series of evaluations. They also used a high-fat diet-induced hypercholesterolaemia mouse model for pharmacodynamic, pharmacokinetic, and bioavailability studies. Thirty minutes after administration, P-4 reached a maximum plasma concentration of 31% oral bioavailability and had a sustained long half-life of 24 h. That study provided a more effective and practical therapeutic option to target uncontrolled hypercholesterolaemia and reduce the risk of CVD, thereby reducing the risk of atherosclerotic CVD and stroke.

Nanocapsules are powerful drug delivery tools that can be used to improve the efficacy of drug therapy and reduce adverse effects. They have various applications in medicine, drug discovery, and clinical treatment, but further research and testing are needed to ensure their safety and efficacy.

Statins are the gold standard in the clinical treatment of AS for primary prevention [181]. Additionally, natural drugs, such as BBR [182,183], artesunate [61], Panax ginseng saponin [184], curcumin [35,185,186], epigallocatechin gallate (EGCG) [187,188], and peptides like PCSK9 antibody inhibitors alirocumab [189] and evolocumab [190] are used to treat high cholesterol and AS. For example, curcumin prevents AS by promoting cholesterol efflux from THP-1 macrophages via the miR-125a-5p/SIRT6 axis and regulating ABCA1 expression [191]. Moreover, EGCG protects ECs from homocysteine-induced EC apoptosis by upregulating the SIRT1/AMPK and Akt/eNOS signalling pathways, thereby attenuating AS pathogenesis [192]. Table 2 summarizes how some drugs may indirectly affect AS by affecting the activity of SIRT proteins or related pathways.

Oral nanoformulations, an emerging approach for AS treatment, offer the promise of more precise drug delivery by improving drug bioavailability through their small size and high surface area properties [18]. However, challenges regarding drug delivery precision, biosafety, and stability remain to be addressed to ensure their effectiveness and safety in clinical applications. Oral nanoformulation development provides new ideas for AS treatment but still requires in-depth research and technological innovation. Table 3 summarizes studies of oral nanoformulations for AS treatment.

## 4. Discussion

AS is a disease of the arterial vasculature that can cause various serious illnesses, such as heart disease and stroke, and its incidence is currently on the rise, with oral medications dominating the treatment strategies [184,195]. The onset of AS usually involves damage to the endothelium, and studies of its pathogenesis have found that the causative factors may include high blood pressure, high serum cholesterol, and smoking, among others [196,197]. AS mechanisms include an inflammatory reaction in the inner layer of the vessel wall due to the uptake of oxidized LDL by ECs [198], and the increased expression of SIRT1 improves vascular endothelial dysfunction and vascular compliance [41]. Additionally, lipid deposition will activate the immune system and cause an inflammatory response [199,200,201]. At this stage, inflammatory cells accumulate in the damaged area, releasing inflammatory mediators, and, under the influence of the inflammatory response, cells within the arterial wall proliferate and produce collagen and elastin fibres, gradually forming plaques [202]. SIRT proteins inhibit the production of inflammatory factors and the development of inflammatory responses by regulating multiple signalling pathways. Some plaques may be unstable and prone to rupture. When a plaque ruptures, platelets in the blood are activated and accumulate on the damaged site, forming a thrombus [88,203,204]. As a plaque grows and blood clots form, arteries narrow and blood flow is impeded [205,206]. Over time, the plaque calcifies and hardens the blood vessel walls, increasing the risk of heart diseases and stroke [207,208]. AS is a gradual process, and its pathogenesis involves the complex action of multiple biological, biochemical, and physiological processes. Since the onset and progression of AS is irreversible, prevention and treatment are crucial.

As AS requires long-term treatment, oral drugs offer obvious advantages due to their rapidity and convenience; therefore, they have become a current research hotspot. Currently, the main drugs used clinically to treat AS are antilipidemic, anticoagulant, and antiplatelet drugs; statins (atorvastatin, rosuvastatin, and simvastatin) [11,113,209], anti-inflammatory drugs, and new lipid-lowering drugs, such as PCSK9 inhibitors [210,211,212,213]. However, AS treatment with conventional drugs is challenged by their high toxicity, low absorption, and inaccurate targeting, which significantly limits their effective clinical application [91,214].

Oral nanoformulations have emerged in recent years as an AS treatment. They can be used to deliver anti-inflammatory drugs, antioxidants, and cholesterol-modulating drugs, among others, to help slow AS progression. Current research on oral nanoformulations focuses on NPs, liposome NPs, SLNs, nanoemulsions, and nanocapsules. Some oral nanoformulations can also be used as medical imaging agents to help doctors better diagnose and monitor AS progression [215].

Oral nanoformulated drugs offer tremendous advantages for AS treatment and diagnosis. They can be surface-modified or designed to enable the targeted delivery of the drug, delivering it accurately to the atherosclerotic plaque and reducing unwanted effects on healthy tissues [216,217]. Dependence on the size and structure of the nano-formulation can be used to achieve controlled drug release, thus ensuring that the drug is released at the right time and place to enhance therapeutic efficacy [218].

However, oral nanoformulations also present some disadvantages. Although they can improve the bioavailability of the drug, the latter must cross biological barriers, such as gastric acid and gastrointestinal mucosa, in the treatment of AS. This may result in some of the drug being ineffective or degraded, reducing its therapeutic effect [219]. AS usually requires the treatment of specific sites of plaque; therefore, precise drug delivery to the target area is required. Oral nanoformulations still face challenges in specific target delivery as they may be absorbed by multiple tissues in vivo rather than being localized to the lesion only. The long-term safety of NPs is unknown, so more research is needed to assess their potential risks [220,221].

Oral nanomedicines have demonstrated great potential in CVD treatment, especially AS. However, developing these formulations faces numerous challenges, including enhancing drug bioavailability, ensuring drug stability, achieving precise targeting, optimizing complex formulation processes, evaluating potential toxicity, and addressing stability issues. Researchers have implemented several strategies to overcome these challenges. These strategies include selecting biocompatible materials, optimizing self-assembly processes to control the NP size, surface modification to enhance targeting and stability, leveraging the EPR effect to increase drug accumulation on disease sites, developing controlled release systems to ensure timely drug release, conducting extensive preclinical studies in animal models to evaluate safety and efficacy, and promoting interdisciplinary collaboration. Continuous research and technological advancements can overcome these difficulties. Future research will focus on improving the safety, stability, and targeting of nanomedicines and conducting clinical trials to validate their efficacy in human patients.

The potential of oral nanoformulated medicines lies in the ability to personalize treatment, tailoring it to the patient’s specific condition and genetic factors. Numerous biological and physiological differences exist between humans and animals that make the successful transition of nanoformulations from the laboratory stage to the clinical therapeutic arena even more challenging. The use of oral nanoformulated drugs in AS treatment is still in the research and development stage. More clinical trials are needed to evaluate their safety and efficacy. Future oral nanoformulations may not be limited to drug delivery but also incorporate various functions, such as inflammation modulation, gene therapy, and tissue engineering, for the integrated treatment of AS. Developing new technologies for monitoring and tracking the distribution and effects of oral nanoformulations in vivo will contribute to a better understanding of their mechanisms of action. Future developments should be devoted to innovation and multidisciplinary collaboration to find more effective treatments. Table 4 summarizes the clinical applications of oral nanoformulations in AS treatment.

## Figures and Tables

**Table 1:** Mechanisms involved in AS pathogenesis.

Pathogenesis	Cell	Relevant Targets	Inflammatory Factors/Therapeutic Pathways	Function	Location of Injury	Therapeutic Drug	References
Endothelial cell damage	Endothelial cell	CAMs (ICAM-1, VCAM-1) E-selectin	TNF-α; IL-1β; NF-κB; NLRP3; ROS	Endothelial cell activation and increased expression of adhesion molecules promote leukocyte adhesion to the vessel wall.	Endothelial layer of the arterial vascular wall	Sal B; Artesunate	[44,61,76,77,78]
Inflammatory response	Macrophages; T lymphocytes; Neutrophils; Monocytes; Dendritic cells	Cytokines; Adhesion molecules; Inflammatory mediators	TNF-α; IL-1β; IL-6; CRP; MCP-1; ICAM-1; VCAM-1; NO	Inflammatory mediators stimulate cell proliferation, migration of smooth muscle cells, and hasten damage to the arterial wall.	Arterial blood vessel wall	Curcumin; Naringin	[35,42,79]
Lipid deposition	Smooth muscle cells; Foam cells; Endothelial cells; Lipid plaques	LDL; ox-LDL-C; CD68; CD36	IL-1; IL-6 IL-8; IL-10; IL-18; ApoE; TLR	Oxidized low-density lipoprotein (LDL) initiates inflammation, promotes phagocytosis by macrophages, and facilitates foam cell formation.	Middle layer of the arterial vascular wall	Quercetin; Atorvastatin combined with nano-selenium	[50,80,81,82]
Smooth muscle cell migration and proliferation	Vascular smooth muscle cells; Vascular endothelial cells; Macrophages	Growth factors (e.g., PDGF); Transforming growth factor-β (TGF-β)	PDGF; TGF-β; IL-8	Activates the proliferation of smooth muscle cells, resulting in the deposition of collagen and the formation of fibrous plaques.	Middle layer of the arterial vascular wall	Rosuvastatin	[64,83,84]
Plaque stability formation	Smooth muscle cells; Inflammatory cells	Collagen in plaques; Elastin; Inflammatory cells	Matrix metalloproteinases (MMPs)	Activation of platelets leads to the rupture of plaque, ultimately resulting in thrombosis.	Patch area	Myricetin; ACE-inhibitor	[67,85,86,87]
Thrombosis	Blood platelet	Platelet activity; Coagulation factors	Thromboxane released by platelets	Forms blood clots that obstruct blood vessels.	Plaque rupture sites within the arterial luminal vessels.	Warfarin	[6,88]
Immune response	T-lymphocytes	T-Lymphocyte activation	CD4+ T helper cells (Th cells); Th17 cells; Regulatory T cells (Treg cells); Memory T cells; Cytotoxic T cells (CD8+ T cells); NK cells; γδ T cells; Interferon-γ (IFN-γ); Interleukin-17 (IL-17)	CD4+ T cells are activated and differentiate into different subpopulations (e.g., Th1 and Th17 subpopulations), releasing pro-inflammatory cytokines, such as interferon-gamma (IFN-γ) and interleukin-17 (IL-17), which are involved in the inflammatory process; Insufficient numbers or dysfunctional Treg cells can create an immune regulation imbalance, which exacerbates the inflammatory response. Memory T cells undergo repeated activation in response to sustained inflammation and immune response. Activation of CD8+ T cells may lead to cytotoxicity; The killing of endothelial cells and macrophages is linked to the activation of NK cells.	Arterial blood vessels	Aspires	[89,90,91]

**Table 2:** Existing drugs that indirectly affect AS by affecting the activity of SIRT proteins or related pathways.

Medicines	Related SIRT Proteins	Clinical Usage	NCT Number	Reference
Atorvastatin	SIRT-1	Assessment of the effect of atorvastatin on coronary atherosclerotic plaque morphology	NCT00576576	[114]
		Effects of atorvastatin in patients with atherosclerosis	NCT00115817	
Rosuvastatin	SIRT-1	Evaluating the effect of rosuvastatin 10–20 mg on the progression of carotid atherosclerosis in Chinese patients	NCT00885872	[110]
		Evaluating the effect of rosuvastatin 20 mg for 76 weeks on coronary atherosclerotic plaque in Chinese patients with coronary heart disease (CHD) hyperlipidaemia compared with baseline	NCT01382277	
Simvastatin	SIRT-1	Anti-inflammatory effects of simvastatin	NCT04638400	[142]
PCSK9 antibody inhibitors	SIRT-3	Effect of PCSK9 inhibitors on coronary microvascular dysfunction in patients with atherosclerotic cardiovascular disease requiring coronary angiographic confirmation of myocardial ischaemia	NCT04338165	[135]
Berberine	SIRT1	Hypolipidemic and vascular effects of nutritional combinations on HIV-infected patients on stable antiretroviral therapy	NCT03470376	[41]
Epigallocatechin gallate (EGCG)	SIRT1	EGCG improves endothelial function	NCT01662232	[192]
Curcumin	SIRT6	Effects of curcumin on diabetic patients with atherosclerotic cardiovascular risk	NCT05753436	[191]
		Effect of short-term supplementation with curcumin and polyphenols on the anti-inflammatory properties of high-density lipoprotein (PSI)	NCT02998918	

**Table 3:** Oral nanoformulations for AS treatment.

Types of Nanoparticles	Nanomedicine	Preparation Methods	Pathways/Targets	Vantage	Disadvantages	Reference
Nanoparticle	Epigallocatechin gallate (EGCG) loaded nanoparticles	Polyelectrolyte composite nanoparticle preparation method	Nrf2/HO-1 pathway; ICAM-1; intercellular cell adhesion molecule-1	Increase drug stability; enhanced efficacy	Causes adverse reactions	[187,188]
	Berberine PLGA-PEG nanoparticles	Nanoprecipitation method	ERK1/2 pathway; Cholesterol efflux from HepG2 cells; ↑ (Upregulates) LDLR; ↓ (Downregulates) PCSK9 expression	Enhance pharmacokinetic properties and expected target outcomes of drugs	Absence of animal studies	[182,183]
	Curcumin nanoparticles	Filming–rehydration method	MIAT/miR-124 pathway; HMGB1-TLRS-NF-κB pathway; LDL-C, TC, TG level	Improving solubility, release performance, and stability of curcumin nanoparticles	Curcumin exhibits poor water solubility and instability during preparation	[35,185,186]
	Diosmin nanoparticles	Emulsion–solvent evaporation method, acid-base neutralization method	TGF-β1; Ang II; TC; TG; HDL-C; PON1	Increased bioavailability, solubility, targeted action	Difficult control over release rate and release site	[193,194]
	pBIN; LApBIN	Dialysis method (pBIN); ultrasonic vibration method (LApBIN)	MCP-1; CCL_2_; TNF-α	Oral adsorption and transport to monocytes, effectively inhibiting inflammation.	There is a lack of sufficient long-term clinical data to assess its long-term efficacy and safety.	[117]
	Rosuvastatin-chitosan nanoparticles	Chitosan gel preparation; o/w emulsion preparation; TPP addition and stirring; nanoparticle separation	NF-κB-p65; IL-6; IL-8; ICAM-1; PECAM-1	Targeted drug delivery, enhanced drug accumulation at the site of lesion, reduced impact on normal tissues.	Stability and efficacy of drug nanocarriers	[111,112]
	Polymer-lipid hybrid nanoparticles of atorvastatin	Single emulsion method, ultrasonication	Akt/FOXO4; α-SMA; SM-MHC; SM22α	Enhancing oral drug absorption, improving bioavailability, enhancing drug efficacy.	Lack of formulation toxicity studies	[112,115]
Nanoemulsion	Rosuvastatin calcium solidified self-nanoemulsifying drug delivery system	Colloidal silica adsorption immobilization technology	NF-κB-p65 passway; IL-6; IL-8; ICAM-1; PECAM-1	Physically stable, conducive to large-scale production; enhanced in vitro dissolution	Stability of self-emulsifying nanoemulsion systems	[111,157]
	Oral nanodispersions stabilized by polysaccharide-protein/protein complexes	Micro jet and ultraviolet irradiation method	Inhibiting inflammatory factors	Improving stability both internally and externally, enhancing mucosal permeability	Individual differences may influence drug efficacy.	[158,159]
Solid lipid nanoparticles	Simvastatin-loaded solid lipid nanoparticles (SLNs)	Ultrasonic emulsification and solidification of nanoparticles	TGF-β/Smad passway; Snail-1; Twist-1; EndMT	Improving oral bioavailability	Preparation and stability of lipid nanoparticles	[140,141]
	Simvastatin solid lipid nanoparticles	High-temperature preparation of nanoemulsions; stabilizing and solidifying nanoemulsions through rapid cooling	TGF-β/Smad passway; Snail-1; Twist-1; EndMT	Reducing dosage administration	Needing more safety assessment and monitoring	[141,143]
Nanosomes	Berberine precursor liposome	The air suspension coating method	KLF16; PPARα	Enhancing the bioavailability of water-insoluble drugs	Challenges in ensuring dose consistency	[132,133]
Nanocapsules	Oral nano liver-targeted anti-PCSK9 drug	Nanocapsule technology	ldl-c; non-HDL-C; apoB; Lp(α)	The first oral nano liver-targeted anti-PCSK9 drug	Complex process; high cost	[178,180]

**Table 4:** Progress in clinical studies on oral drugs for AS treatment.

Drug Type	Medications	Mechanisms	Current Status and Future Prospects	References
Statin Drugs	Atorvastatin	Akt/FOXO4 pathway; VSMCs phenotypic modulation; prevention and treatment of atherosclerosis	Polymer and lipid-polymer hybrid oral nanoparticles loaded with atorvastatin Chitosan-modified PLGA atorvastatin-curcumin conjugated (AT-CU) nanoparticles Hyaluronic acid-conjugated atorvastatin nanoparticles Nanopreparations of atorvastatin have been successfully developed and demonstrated promising results in animal experiments. They are expected to be applied clinically in the near future.	[11,113,115,116]
	Rosuvastatin	↓ NF-κB-p65; ↓ expression of IL-6, IL-8, ICAM-1, and PECAM-1; slowing the progression of atherosclerosis	Rosuvastatin-chitosan oral nanoparticles Rosuvastatin calcium solidified self-nanoemulsifying drug delivery system Oral nanomedicines of rosuvastatin, such as nanoparticles and nanoemulsions, have achieved breakthrough progress and are expected to be applied clinically soon.	[111,112,157]
	Simvastatin	↓ Oxidative stress, TGF-β/Smad signalling ↓, inactivation of Snail-1 and Twist-1; ↓ EndMT induced by povpc; ↓ atherosclerosis	Simvastatin (SV) lipid nanoparticles (SLNs) Simvastatin oral solid lipid nanoparticles Simvastatin nanoprodrugs and their fibronectin-targeted co-delivery systems Oral nanomedicines of simvastatin have shown promising results in animal experiments and are expected to be used clinically in the near future.	[140,141,143,209]
Peptide Drugs	PCSK9 antibody inhibitors	↓ LDL, apoB, Lp(a); ↓ risk of atherosclerosis	Oral nano-liver small molecules targeting PCSK9 Injectable biomimetic nanoliposomes loaded with PCSK9 inhibitor evolocumab Injectable PCSK9 nanoliposomes and oral nanocapsules are expected to be successfully applied in clinical treatment in the future.	[136,178,180]
Natural Drugs	Berberine	↑ Expression of KLF16; ↑ PPARα; ↑ interaction of KLF16 and PPARα; ↓ atherosclerosis	Berberine precursor liposomes Berberine-encapsulated PLGA–PEG nanoparticles Related oral nanomedicines of berberine have shown good therapeutic effects in in vivo and in vitro experiments and are expected to be applied clinically.	[132,133,182,183]
	1,8-Cineole (CIN)	↓ Lipid parameters, ↓ expression of inflammatory factors and proteins	Polysaccharide–protein/protein complex stabilized oral nanoemulsions Nanoemulsions improve drug stability in the body, prolong GIT residence time, and enhance bioavailability. With advancements in research, they are expected to be applied clinically.	[158,159]
	Curcumin	MIAT/miR-124, HMGB1-TLRS-NF-κB signalling pathway; ↓ serum LDL-C, TC, TG levels; ↓ atherosclerosis	Curcumin-loaded nanoparticles Curcumin nanoparticles significantly reduce atherosclerotic lesions and are more effective in stabilizing vulnerable plaques. They are expected to be an option for clinical use after long-term stability and safety trials.	[35,185,186]
	Epigallocatechin gallate (EGCG)	Mediates Nrf2/HO-1 pathway; ↓ ICAM-1 and PECAM-1; ↓ monocyte adhesion → treatment of atherosclerosis	Chitosan (CS) and polyaspartic acid (PAA) nanoparticles loaded with EGCG Nanoparticles increase stability in the human stomach and intestines, promote drug absorption, and improve EGCG’s therapeutic efficacy against atherosclerosis.	[187,188]

## Data Availability

Not applicable.
